# 

**Published:** 1879-06

**Authors:** 


### CONTENTS.

ORIGINAL.

ART. I.—Malignancy in Tumors. By P. W. Van Peyma, M. D., ........ 401

ART. II.—Proceedings of the Buffalo Medical Association. Paper by Prof. James P. White, M. D., ........ 411

ART. III.—A Renal Calculus. Service of Dr. C. Diehl. Reported by Fred. Peterson, ... 416

MISCELLANEOUS.

Mechanical Treatment of the Hip, Knee and Ankle-joints, by a simple and efficient method. The Physiological method with cases and illustrations, ........ 418

EDITORIAL.

The Convention of Medical Colleges, ........ 433

The Association of American Medical Editors, 433

The American Medical Association ........ 434

Transactions of Gynecological Society, 1878, .. 435

State Association of Druggists, ........ 435

Fourth Annual Meeting of the State Society of Arkansas, ........ 436

Lactopeptine, ........ 436

Books Reviewed, ........ 436

Books Received, ........ 439

Pamphlets, ........ 440

